# Autoantibodies neutralizing type I IFNs in patients with fulminant herpes simplex virus hepatitis

**DOI:** 10.1084/jem.20251760

**Published:** 2025-12-05

**Authors:** Adrian Gervais, Astrid Marchal, Soraya Boucherit, Anthony Abi Haidar, Lucy Bizien, Ahmet Yalcinkaya, Ella Sandström, Xiao-Fei Kong, Emmanuel Jacquemin, Olivier Bernard, Dominique Debray, Florence Lacaille, Philippe Ichai, Cigdem Arikan, Etienne Javouhey, Bertrand Roquelaure, Frédéric Gottrand, Francesca Trespidi, Veronica Codullo, Lorenzo Cavagna, Nicolas Schleinitz, Mohamed Bousfiha, Naima Amenzoui, Ahmed Aziz Bousfiha, Sofie E. Jørgensen, Nanna Mørk, Trine H. Mogensen, Paul Bastard, Anne Puel, Alessandro Borghesi, Jody A. Rule, William M. Lee, Nils Landegren, Aurélie Cobat, Jean-Laurent Casanova, Emmanuelle Jouanguy

**Affiliations:** 1 Laboratory of Human Genetics of Infectious Diseases, Necker Branch, Institut National de la Santé et de la Recherche Médicale (INSERM) U1163, Necker Hospital for Sick Children, Paris, France; 2 Paris Cité University, Imagine Institute, Paris, France; 3 Department of Medical Biochemistry and Microbiology, Science for Life Laboratory, Uppsala University, Uppsala, Sweden; 4 Department of Medical Biochemistry, Hacettepe University Faculty of Medicine, Ankara, Türkiye; 5 Division of Digestive and Liver Diseases, Department of Internal Medicine, UT Southwestern Medical Center, Dallas, TX, USA; 6 McDermott Center for Human Growth and Development, UT Southwestern Medical Center, Dallas, TX, USA; 7 Pediatric Hepatology and Liver Transplantation Unit, National Reference Center for Inflammatory Diseases of the Bile Ducts and Autoimmune Hepatitis, FILFOIE, ERN RARE LIVER, Bicêtre Hospital, AP-HP, University Paris-Saclay, Le Kremlin-Bicêtre, France and Inserm UMR_S 1193, University Paris-Saclay, Orsay, France; 8 Pediatric Liver Unit, National Competence Centre for Inflammatory Diseases of the Bile Ducts and Autoimmune Hepatitis, FILFOIE, ERN RARE LIVER, Assistance Publique-Hôpitaux de Paris (AP-HP)-Necker-Enfants Malades Hospital, University of Paris, Paris, France; 9 Department of Pediatric Gastroenterology-Nutrition and Pediatric Liver Unit, Necker-Enfants Malades Hospital, University of Paris, Paris, France; 10 Liver Intensive Care Unit, Centre Hépato-Biliaire, AP-HP, Hôpital Paul-Brousse, Université Paris-Saclay, Inserm Research Unit 1193, Villejuif, France; 11 Koc University School of Medicine, Pediatric Gastroenterology and Hepatology, Organ Transplantation and Research Center, Koc University Research Center for Translational Medicine (KUTTAM), Istanbul, Turkey; 12 Pediatric Intensive Care Unit, Hôpital Femme Mère Enfant, Hospices Civils de Lyon, Lyon, France; 13 Multidisciplinary Pediatrics Department, Timone Enfants Hospital, Assistance Publique-Hôpitaux de Marseille (AP-HM), Marseille, France; 14 Pediatric Gastroenterology Hepatology and Nutrition Department, University of Lille, CHU Lille, Lille, France; 15 Host-Pathogen Group and Neonatal Intensive Care Unit, San Matteo Research Hospital, Pavia, Italy; 16 Division of Rheumatology, San Matteo Research Hospital, Pavia, Italy; 17 Department of Internal Medicine and Therapeutics, University of Pavia, Pavia, Italy; 18 Internal Medicine Department, La Timone AP-HM, Aix-Marseille Université, Marseille, France; 19 Pediatric Infectious Diseases and Clinical Immunology Department, Children’s Hospital, CHU Ibn Rochd, Casablanca, Morocco; 20 Laboratory of Clinical Immunology, Infection and Autoimmunity (LICIA), Faculty of Medicine and Pharmacy, Hassan II University Casablanca, Casablanca, Morocco; 21 Department of Biomedicine, Aarhus University, Aarhus, Denmark; 22 Department of Infectious Diseases, Aarhus University Hospital, Aarhus, Denmark; 23 St. Giles Laboratory of Human Genetics of Infectious Diseases, Rockefeller Branch, Rockefeller University, New York, NY, USA; 24 Pediatric Hematology-Immunology and Rheumatology Unit, Necker Hospital for Sick Children, AP-HP, Paris, France; 25 Neonatal Intensive Care Unit, University Hospitals of Geneva, Geneva, Switzerland; 26 Department of Paediatrics, Gynaecology and Obstetrics, University of Geneva, Geneva, Switzerland; 27 School of Life Sciences, Swiss Federal Institute of Technology, Lausanne, Switzerland; 28 Howard Hughes Medical Institute, New York, NY, USA; 29 Department of Pediatrics, Necker Hospital for Sick Children, Paris, France

## Abstract

Fulminant viral hepatitis (FVH) is a devastating condition caused by hepatotropic viruses such as hepatitis A virus (HAV), hepatitis B virus (HBV), and HSV-1/2. We studied 149 FVH patients (73 males and 76 females, aged 1–76) for blood autoantibodies (auto-Abs) neutralizing type I interferons (IFNs; IFN-α2, -β, -ω). Six of 16 (37.5%) HSV-triggered FVH patients carried such auto-Abs on admission, including three with a previously known autoimmune disease. These patients contrasted with 133 HAV- (*n* = 46) or HBV-triggered (*n* = 87) patients, none of whom had such detectable auto-Abs. Odds ratios for HSV-triggered FVH in individuals with auto-Abs ranged from 35.3 (95% CI: 13.0–96.2; P < 10–7) for those neutralizing only 100 pg/ml IFN-α/ω to 1,895 (CI: 448.5–8,002; P < 10–12) for those neutralizing both IFN-α and IFN-ω at 10 ng/ml. Over one third of HSV-triggered FVH cases in this international cohort were due to preexisting auto-Abs. This finding highlights auto-Abs against type I IFNs as a major determinant of HSV-FVH and paves the way for targeted preventive or therapeutic interventions.

## Teaser

Fulminant viral hepatitis (FVH) is a devastating condition caused by hepatotropic viruses. In 149 international FVH patients, auto-Abs neutralizing type I interferons are found in 37.5% of HSV-triggered cases and are absent in hepatitis A and B virus–triggered cases, increasing the risk of HSV-FVH by >1,800-fold.

## Introduction

Fulminant viral hepatitis (FVH) is a severe, rapidly progressing form of liver failure characterized by massive hepatocyte death and hepatic encephalopathy, requiring immediate intensive care (Jouanguy, 2020; Li et al., 2022). Mortality due to FVH is high, at about 80% in the absence of liver transplantation (Lemon et al., 2018). The long-term outcome of patients after transplantation is also poor, with a mortality of ∼35% in the first year after transplantation. FVH can be triggered by various hepatotropic viruses, principally the hepatitis A and B viruses (HAV, HBV), and more rarely, herpes simplex virus (HSV)-1 and HSV-2 (Farci et al., 1996; Liu et al., 2001). The prevalence and incidence of FVH in unvaccinated populations are unknown. Vaccination campaigns against HAV and HBV have significantly decreased the incidence of FVH worldwide (Cervio et al., 2011; Nelson et al., 2011). About 10–45% of acute liver failure (ALF) cases are currently due to viral infections (Stravitz and Lee, 2019), and the incidence of ALF is estimated at ∼1/10^−5^ annually in the USA (Weiler et al., 2020) and other Western countries. The annual incidence of FVH may therefore be estimated at between 1 and 5 cases per million of infected individuals in Western countries.

HAV and HBV are the main agents of FVH, accounting for 31% and 25% of all FVH cases, respectively, with other viruses less frequently involved (Gimson et al., 1983; Ichai and Samuel, 2011). About 0.1% of acute HBV infections progress to FVH in unvaccinated adults, and FVH occurs in 0.015% to 0.5% of acute HAV cases in unvaccinated children (Farooq et al., 2019; Manka et al., 2016). The incidence of hepatitis E virus (HEV)–triggered FVH is less well known, but this condition is a significant cause of concern in endemic regions, especially in pregnant women (Braira Wahid, 2022). FVH has been reported even more rarely in patients co-infected with HBV/hepatitis D virus, HAV/HEV, or hepatitis C virus/another virus, or infected with HHV-6 or enteroviruses (Charnot-Katsikas et al., 2016; Farci et al., 1996; Farci and Niro, 2012; Liaw et al., 2004; Vento et al., 1998). Finally, HSV-induced FVH has been estimated to account for about 1% of all causes of FVH in the USA (Vinholt Schiødt et al., 2003). FVH is, thus, rare, and mostly driven by HAV and HBV or more rarely by HSV-1 or HSV-2 (Vinholt Schiødt et al., 2003). The annual incidence of FVH worldwide is not precisely known, but it can be estimated to range from several thousands to over a hundred thousand cases annually.

The pathogenesis of FVH remains unknown, as most individuals infected with the viruses concerned do not develop FVH. Its rarity, and its sporadic, as opposed to epidemic, nature suggest that the viruses involved are unlikely to be particularly virulent (Ajmera et al., 2011). A few case-control association studies have suggested that common variants of *TIM1* (Kim et al., 2011) and *CXCL16* (Ajmera et al., 2019) increase the risk of FVH by factors of 1.3 and about 1.6-fold, respectively. Moreover, variants of genes encoding intracellular viral sensors or other molecules involved in type I interferon (IFN) immunity were found in 10 out of 24 HEV-FVH patients tested but not in the control group (Saadat et al., 2024). Furthermore, reports of multiplex and/or consanguineous families have suggested a possible contribution of monogenic inborn errors of immunity (IEIs) (Casanova, 2025). For example, autosomal recessive (AR) deficiencies of the IL-18 binding protein (IL-18BP) and the IL-10 receptor B subunit have been reported in patients with HAV-FVH (Abd Elaziz et al., 2025; Belkaya et al., 2019; Korol et al., 2023). These patients may have an immunological feature in common: the unleashing of type II IFN activity via an enhancement of IL-18 activity or a decrease in IL-10 activity, leading to excessive macrophage activation in the virus-infected liver.

Other severe viral infections of isolated organs, such as viral encephalitis and pneumonia, have been explained by rare IEI impairing the production of or response to type I IFNs (Casanova, 2025; Casanova and Abel, 2022). Moreover, autoantibodies (auto-Abs) neutralizing type I IFNs (AAN-I-IFNs) have been implicated in disease in larger proportions of patients. They underlie 5–20% of cases of critical COVID-19 pneumonia (Abers et al., 2021; Acosta-Ampudia et al., 2021; Akbil et al., 2022; Arrestier et al., 2022; Bastard et al., 2021a; Bastard et al., 2020b; Busnadiego et al., 2022; Carapito et al., 2022; Chang et al., 2021; Chauvineau-Grenier et al., 2022; Credle et al., 2022; Eto et al., 2022; Frasca et al., 2022; Goncalves et al., 2021; Grimm et al., 2022; Hale, 2023; Hansen et al., 2023; Koning et al., 2021; Lamacchia et al., 2022; Lemarquis et al., 2021; Mathian et al., 2022; Meisel et al., 2021; Petrikov et al., 2022; Philippot et al., 2023; Pons et al., 2023; Raadsen et al., 2022; Savvateeva et al., 2021; Schidlowski et al., 2022; Simula et al., 2022; Solanich et al., 2021; Soltani-Zangbar et al., 2022; Su et al., 2022; Troya et al., 2021; van der Wijst et al., 2021; Vanker et al., 2023; Vazquez et al., 2021; Wang et al., 2021; Ziegler et al., 2021), 5% of cases of severe influenza pneumonia (Zhang et al., 2022), 20% of cases of severe Middle East respiratory syndrome pneumonia (Alotaibi et al., 2023), about a third of severe adverse reactions to the attenuated live measles and yellow fever virus vaccines (Bastard et al., 2021b), ∼40% of West Nile virus (WNV) encephalitis cases (Gervais et al., 2023), ∼10% of the most severe forms of tick-borne encephalitis (Gervais et al., 2024c), and most cases of the rarer Powassan virus, Usutu virus, and Ross River virus diseases studied (Gervais et al., 2024a). These auto-Abs are present in the general population, with a prevalence increasing from 0.3–1% in individuals under 65 years of age to 4–7% in those over 65 years of age (Bastard et al., 2024).

AAN-I-IFNs also seem to predispose to some infections with some herpes viruses. Indeed, patients with autoimmune polyendocrinopathy syndrome type 1, most if not all of whom carry these auto-Abs, have an increased risk of recurrent or severe mucocutaneous disease due to HSV or varicella zoster virus (VZV) (Hetemäki et al., 2021). Likewise, these auto-Abs increased the risk of recurrent herpesvirus infections in patients mutated in recombination activating gene (*RAG)* genes (Delmonte et al., 2020; Dutmer et al., 2015), especially VZV and cytomegalovirus (CMV), or patients with severe COVID-19 (Busnadiego et al., 2022). Moreover, monoclonal antibody therapy targeting the IFN alpha receptor 1 (IFNAR1) elicited HSV-2 acute hepatitis in a patient with systemic lupus erythematosus (SLE) (Larsen et al., 2024), while AAN-I-IFNs were found in a patient with HSV-2 acute hepatitis (Martinot et al., 2023). Both were hospitalized and treated before liver failure occurred. In this light, we hypothesized that such auto-Abs may also underlie FVH, at least in some patients, particularly upon infection with HSV.

## Results

### A cohort of 149 patients with FVH

**Figure 1.**
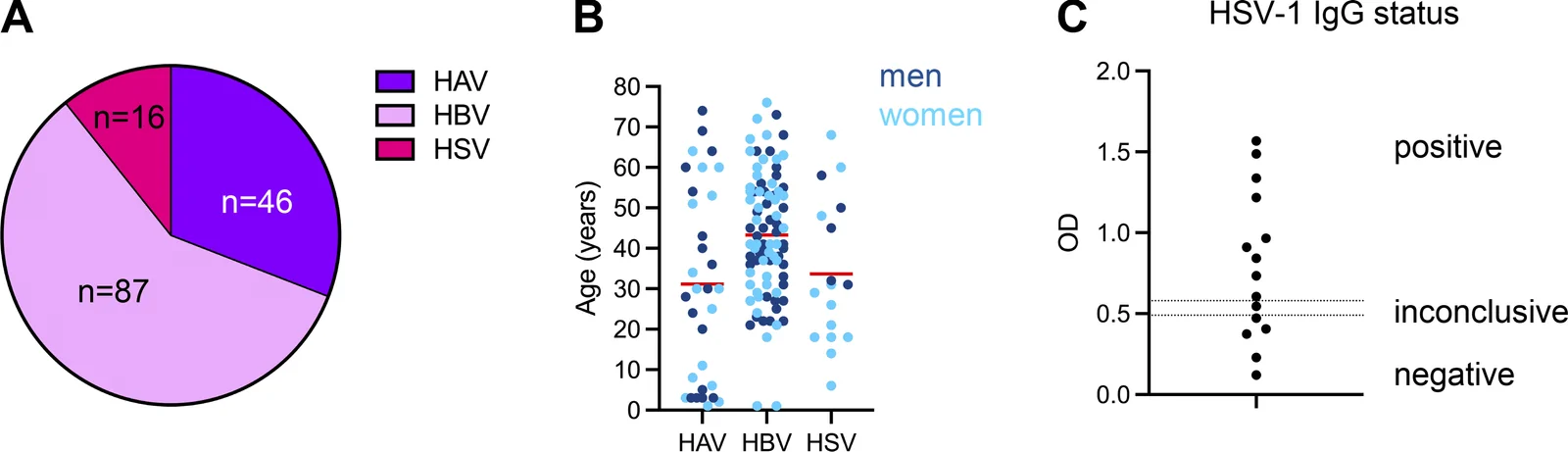
**Description of the FVH cohort. (A)** Distribution of the viruses implicated in FVH in these FVH patients. **(B)** Sex distribution of the FVH patients according to the infecting virus. **(C)** Anti-HSV-1 IgG titers in the plasma of HSV-FVH patients, as determined by ELISA.

We analyzed 149 FVH patients, all hospitalized: 46 with HAV, 87 with HBV, and 16 with HSV (HSV-1: 9; HSV-2: 5; both: 2). Mean ages were 38.6 years overall (range 1–76), with subgroup means of 31·1 (HAV), 43.3 (HBV), and 29.4 (HSV) years (Fig. 1 A and Table 1). Males comprised 48.7% overall: 50% in HAV, 51.7% in HBV, and 35% in HSV groups (Fig. 1 B and Table 1). Infections occurred from 1980 to 2024. Patients came from the USA (*n* = 102), France (31), Morocco (12), Italy (2), and Turkey (2). Viral triggers were confirmed in all HAV/HBV cases via virus-specific IgM and/or IgG. HSV infection was confirmed by anti-HSV IgM/IgG in nine patients, and PCR in blood/liver in the other seven patients. Among HSV cases, five had primary infections (based on anti-HSV-1 IgG on admission), 10 had prior infections, and one case was inconclusive (Fig. 1 C). Among all patients, 35/149 (23.5%) died, including 2/46 (4.3%) HAV, 29/87 (33.3%) HBV, and 4/16 (25.0%) HSV patients (Table 1). In other words, mortality was comparable in non-HSV-FVH (31/133, 23%) and HSV-FVH (4/16, 25%). None of the 149 patients were found to carry biallelic or X-linked mutations known to underlie FVH, impair the production of or the response to type I IFNs (including AR TLR3, IRF7, IFNAR1/2, IRF9, STAT1, STAT2 deficiencies, autosomal dominant (AD) IRF3 deficiency, and X-linked recessive TLR7 and NEMO deficiencies) (Abolhassani et al., 2022; Andersen et al., 2015; Asano et al., 2021; Audry et al., 2011; Bastard et al., 2022; Bastard et al., 2020a; Bravo García-Morato et al., 2019; Duncan et al., 2022; Dupuis et al., 2003; Hambleton et al., 2013; Hernandez et al., 2019; Hernandez et al., 2018; Thomsen et al., 2019; Zhang and Casanova, 2015; Zhang et al., 2007), or the production of AAN-I-IFNs (including AR AIRE, RAG1/2, RELB, and NIK deficiencies, a specific form of AD NFKB2 deficiency, and X-linked recessive FOXP3 deficiency) (Bastard et al., 2021c; Bosticardo et al., 2021; Le Voyer et al., 2023; Lemarquis et al., 2021; Meager et al., 2006; Rosenberg et al., 2018; Schidlowski et al., 2022; Sjøgren et al., 2022).

**Table 1.** General demographic data of the FVH cohort

​	HAV	HBV	HSV	Total
Mean age (years)	31.1	43.3	29.4	38.6
Male proportion (%)	50.0	51.7	35.0	48.7
Mortality (%)	4.3	33.3	25.0	23.5

### Auto-Abs neutralizing IFN-α2, -β, and/or -ω in FVH patients

**Figure 2.**
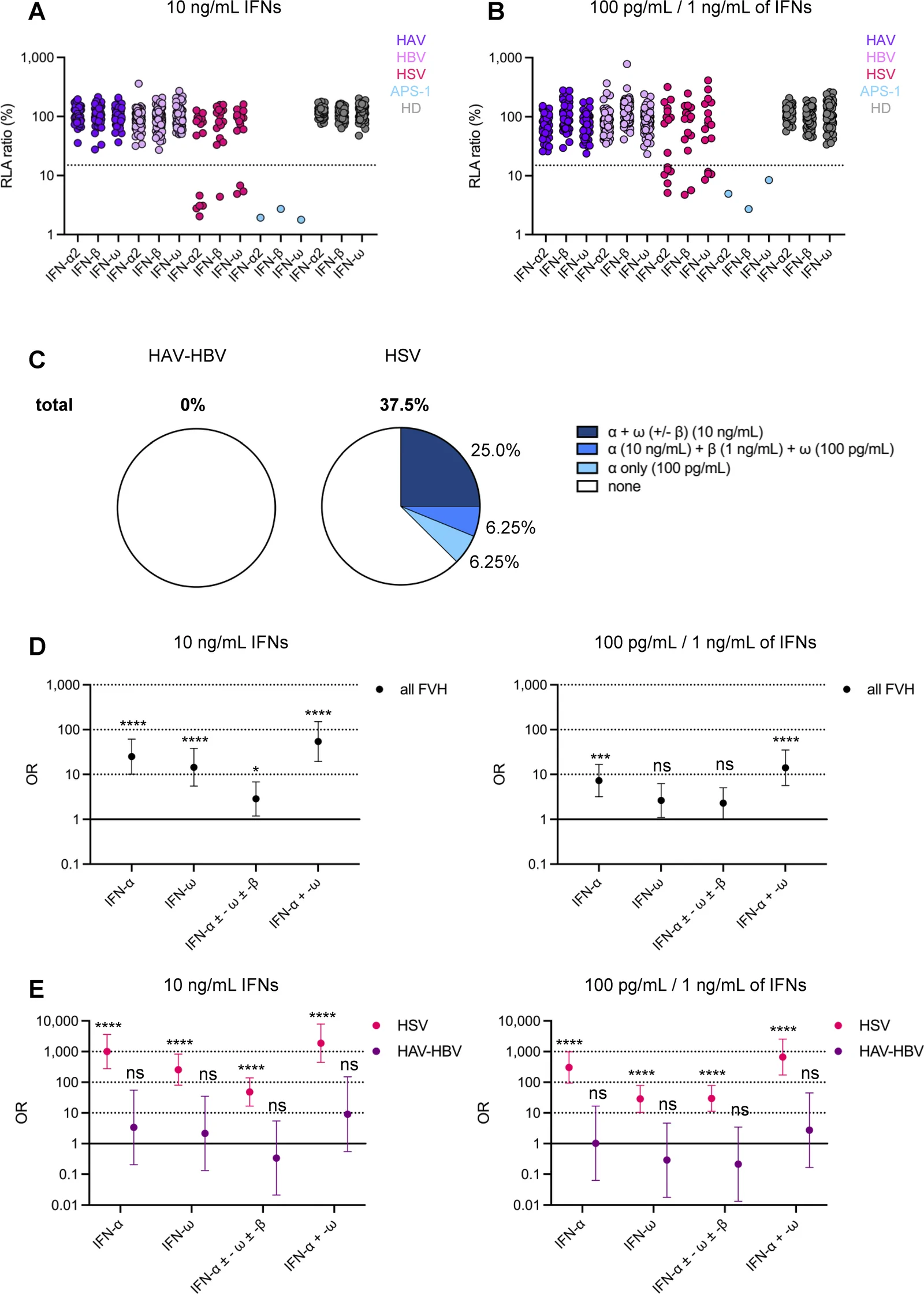
**AAN-I-IFNs in patients with FVH and associated OR. (A)** Luciferase-based neutralization assay to detect auto-Abs neutralizing 10 ng/ml IFN-α2, IFN-ω, or IFN-β. Samples with a RLA <15% are considered neutralizing. **(B)** Luciferase-based neutralization assay to detect auto-Abs neutralizing 100 pg/ml IFN-α2 or IFN-ω or 1 ng/ml IFN-β. **(C)** Proportion of type I IFNs neutralized in the patients, in individuals with HAV-HBV FVH and in individuals with HSV-FVH. **(D)** OR for the presence of AAN-I-IFNs in all individuals with FVH, relative to the general population, with adjustment for age by logistic regression. The horizontal bars indicate the upper and lower limits of the 95% CI. IFN-α, auto-Abs neutralizing IFN-α2 (regardless of their effects on other IFNs); IFN-ω, auto-Abs neutralizing IFN-ω (regardless of their effects on other IFNs); IFN-α ± ω ± β, auto-Abs neutralizing IFN-α2 and/or IFN-ω and/or IFN-β; IFN-α + ω, auto-Abs neutralizing both IFN-α2 and IFN-ω. **(E)** OR for the presence of AAN-I-IFNs in individuals with HSV-FVH vs. HAV/HBV-FVH.

**Figure S1.**
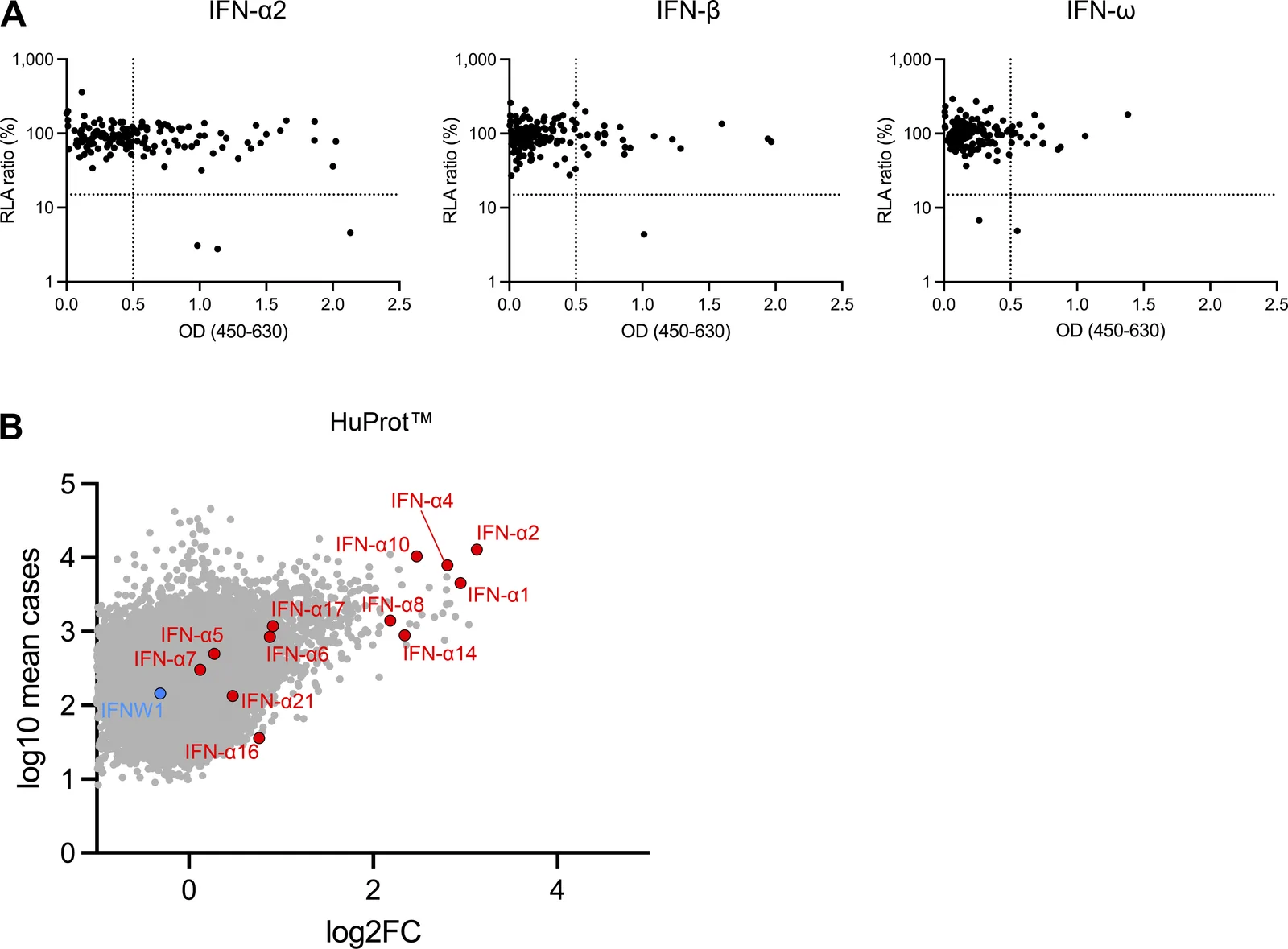
**Detection of auto-Abs by ELISA and HuProt. (A)** Correlation between ELISA and neutralization assay results for the detection auto-Abs. **(B)** Detection of auto-Abs by HuProt in the six HSV-triggered FVH with AAN-I-IFNs FVH vs. healthy donors.

Using a previously described luciferase-based neutralization assay, we tested 1:10 dilutions of serum/plasma for the ability to neutralize high (10 ng/ml) or low (100 pg/ml) levels of non-glycosylated IFN-α2 and IFN-ω, and high (10 ng/ml) or intermediate (1 ng/ml) levels of glycosylated IFN-β. Samples were collected from 51% of patients within 30 days of symptom onset, and the rest between 2 mo and 48 years after FVH. Incubation periods vary from 2 to 14 days (HSV-1/2) to 5 to 180 days (HAV/HBV). Among HSV-FVH patients, five (31%) had auto-Abs neutralizing 10 ng/ml type I IFNs: two targeted IFN-α2 only, two both IFN-α2 and IFN-ω, and one of all three IFNs (Fig. 2 A). At lower concentrations, these five plus one additional patient neutralized IFN-α2, totaling six HSV-infected patients (37.5%) with AAN-I-IFNs (Fig. 2 B). These auto-Abs were also detected by ELISA and HuProt microarray in four of the six cases (Fig. S1, A and B). Auto-Abs were present in 2.8% of FVH deaths vs. 4.4% of survivors overall, and in 25% of HSV-FVH deaths vs. 42% of HSV-FVH survivors. Of the six HSV-FVH patients with auto-Abs, four had primary infections, two had reactivations. By contrast, no HAV/HBV-FVH patients had neutralizing auto-Abs. Even a single IFN-neutralizing auto-Ab may impair anti-HSV immunity, as seen in other viral diseases. Overall, AAN-I-IFNs were found in 6 (37.5%) of 16 HSV-FVH patients, but in none of 133 HAV/HBV-FVH cases (P < 10^−6^) (Fig. 2 C).

### Clinical descriptions of the patients with AAN-I-IFNs

P1 was an 18-year-old Italian woman with HSV-2 infection and a prior diagnosis of HLA-B27 spondyloenthesoarthritis and seronegative rheumatoid arthritis. Treated with anti-TNF and methotrexate, she was hospitalized for HSV-2–associated hepatitis and pancreatitis. Acyclovir therapy lasted 6 mo, until serum HSV-2 DNA cleared. After infection, she experienced cutaneous HSV-2 recurrences, hand and foot blisters, and elevated liver enzymes, all resolving with antivirals. P2, a 68-year-old Italian woman with nephrotic syndrome, xerophthalmia, oral lichen, and recurrent CMV/VZV infections, developed HSV-1 FVH and later hepatic HSV reactivation, both treated with acyclovir. She was subsequently diagnosed with undifferentiated arthritis and subclinical hypothyroiditis, managed with hydroxychloroquine. P3 was a 21-year-old American woman with HSV-1 and preexisting SLE, treated with azathioprine. This case mirrors previous reports of SLE patients with AAN-I-IFNs, who exhibited lower disease activity and increased susceptibility to viral infections (Mathian et al., 2022). Despite acyclovir, she died from FVH. P4, a 50-year-old American man without prior autoimmune or infectious history, survived HSV-1 infection after acyclovir treatment. P5 was a 58-year-old American man with chronic sinusitis who survived HSV-1 infection thanks to liver transplantation. P6 was an 18-year-old French woman with HSV-2, successfully treated with acyclovir. She later developed anal herpes and showed chronic inflammation and antinuclear antibodies, although no autoimmune disease was identified. All six patients had FVH prior to the COVID-19 pandemic. The subsequent SARS-CoV-2 infection status was only documented for P6; she had a symptomatic SARS-CoV-2 infection in 2021, which did not require hospitalization. Notably, half the patients were young (18–21 years old), contrasting with the typical age-related prevalence of AAN-I-IFNs, and half had previous or simultaneous diagnosis of an autoimmune condition (Table 2).

**Table 2.** General clinical characteristics of the six patients with HSV-FVH and AAN-I-IFNs

Patient	Age	Sex	Virus	FVH outcome	Autoimmune history
P1	18	F	HSV-2	Survival	HLA-B27 spondyloenthesoarthritis, seronegative rheumatoid arthritis
P2	68	F	HSV-1	Survival	Arthritis, antinuclear antibodies, anti-smooth muscle antibodies
P3	21	F	HSV-1	Death	Systemic lupus erythematous
P4	50	M	HSV-1	Survival	-
P5	58	M	HSV-2	Survival	-
P6	18	F	HSV-2	Survival	-

### Risk of FVH in individuals with AAN-I-IFNs

We previously assessed AAN-I-IFNs prevalence in the French population via neutralization assays on 34,159 healthy adults (20–100 years) and 2,272 children (0–19 years). By comparing FVH patients with this population, and adjusting for age, we estimated FVH risk conferred by AAN-I-IFNs. In the full FVH cohort, auto-Abs neutralizing 10 ng/ml of at least one type I IFN were linked to increased FVH risk (odds ratio [OR] = 2.8, 95% CI: 1.2–6.8, P = 0·04). The association was borderline for auto-Abs neutralizing 100 pg/ml (OR = 2.3; 95% CI: 1.0–1.0, P = 0·07) (Fig. 2 D). Stratifying by virus, AAN-I-IFNs were strongly associated with HSV-FVH, with ORs from 52.7 (95% CI: 17.8–155.9; P < 10^−7^) for any neutralizing auto-Abs to 1,895 (CI: 448.5–8,002; P < 10^−12^) for auto-Abs neutralizing both IFN-α2 and IFN-ω at 10 ng/ml (Fig. 2 E). These findings support prior evidence linking broader and stronger neutralization of type I IFNs with severe viral disease.

### Longitudinal samples

**Figure 3.**
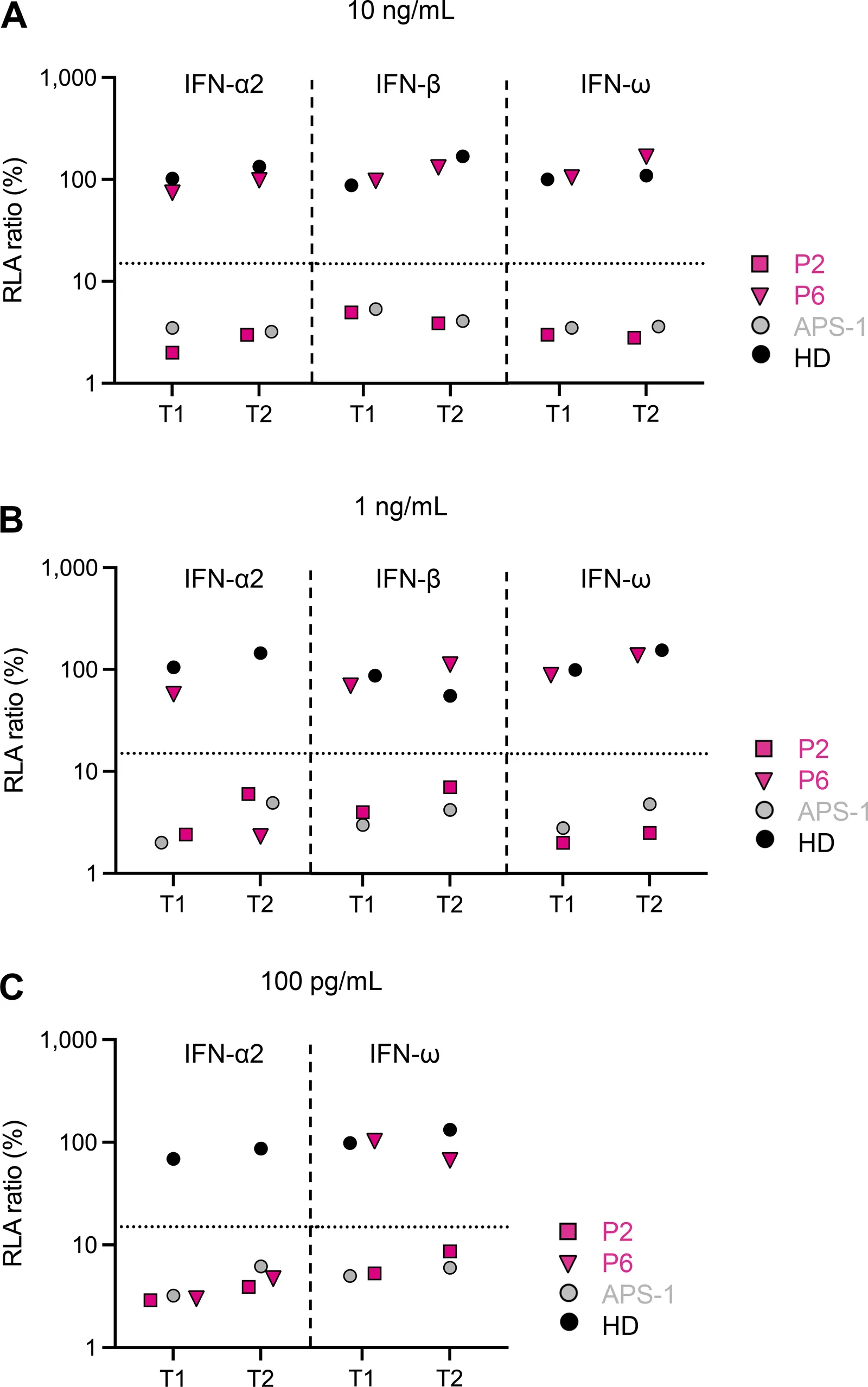
**AAN-I-IFNs in longitudinal samples, as determined with the luciferase-based neutralization assay in P2 and P6 samples. (A–C)** Plasma samples were diluted 1:10 and incubated with 10 ng/ml (A), 1 ng/ml (B), or 100 pg/ml (C) IFNs. Plasma samples from the APS-1 patients and the healthy donor were not longitudinal and were only used as control for type I IFNs neutralization. APS-1, autoimmune polyendocrinopathy syndrome type 1.

Samples were collected from the six patients with AAN-I-IFNs 1 day (P1, P2, and P5), 7 days (P3), 2 days (P4), and 5 years (P6) after the onset of FVH. We studied the neutralization of type I IFNs in longitudinal plasma samples for two patients (P2 and P6, Fig. 3, A–C). We had two samples for P2, obtained 3 years apart (one collected the first day of HSV infection, and another collected 3 years later). Both samples were able to neutralize 10 ng/ml IFN-α2, -β, and -ω, demonstrating the stability of these pathogenic immunoglobulins. We had two samples taken 6 years apart for P6 (one collected 5 years after HSV infection, and the other collected 11 years later). Interestingly, we found that the initial sample (closer to the episode of FVH) contained auto-Abs neutralizing only 100 pg/ml IFN-α2, whereas the subsequent sample was able to neutralize this IFN at concentrations up to 1 ng/ml. Thus, these auto-Abs do not disappear over time but their amounts and/or affinity for their target may vary over time, consistent with previous reports, showing that auto-Abs do not disappear but instead diversify and become more potent with time (Fernbach et al., 2024).

## Discussion

We were able to clarify the cause and mechanism of HSV-triggered FVH in over a third of the cases studied. These patients had AAN-I-IFNs at the time of HSV-1 or HSV-2 infection of the liver, suggesting that defective type I IFN–dependent immunity was the cause of HSV-triggered FVH. The arguments for causality are as follows. First, the prevalence of AAN-I-IFNs is much higher in cases than in healthy individuals of the same age group, with an OR of 319, for AAN-I-IFNs neutralizing only 100 pg/ml IFN-α2, and >1,800, for AAN-I-IFNs neutralizing both IFN-α2 and IFN-ω at 10 ng/ml. This impact is like that of highly penetrant monogenic lesions. Second, the AAN-I-IFNs were detected on the day of admission in four of the six patients, implying that they were present before viral infection. This was probably also the case for the other two patients, for whom plasma samples collected at admission were not available. Indeed, we showed in another study that the neutralization of type I IFNs requires multiple rounds of Ab maturation in the germinal centers (Sokal et al., 2021). Third, these AAN-I-IFNs have been previously shown to cause various cerebral and respiratory viral diseases, in unprecedented proportions, best illustrated by the 40% of cases of WNV encephalitis that can be explained by these AAN-I-IFNs (Gervais et al., 2023). Our findings add the liver to the list of organs known to require type I IFNs to fend off viruses. Fourth, these AAN-I-IFNs were found only in patients with HSV-triggered FVH, not in those with FVH triggered by HAV or HBV, suggesting a virus-specific requirement for their impact on the outcome of hepatitis. Nevertheless, other, hitherto unknown factors probably contributed to the pathogenesis of HSV-FVH.

These findings also clarify the mechanism of disease in patients with HSV-induced FVH. They suggest that type I IFNs are required for protective immunity in the liver upon infection with HSV-1 and HSV-2, whereas they seem to be redundant for controlling infection with other liver-tropic viruses, such as HAV and HBV. These findings suggest that type I IFN therapy may be useful, in addition to antiviral therapies, in patients with HSV-triggered FVH, especially if given early in infection. Patients with auto-Abs against IFN-ω may benefit from early treatment with IFN-α2 or -β, while patients with auto-Abs against IFN-α2 may benefit from early IFN-β therapy. Other therapeutic options could include the use of decoys that prevent type I IFN neutralization (Groen et al., 2025) or the use of chimeric auto-Ab receptor T-cells specifically targeting AAN-I-IFNs producing B cells (Peng et al., 2025). The contribution of type I IFN to protective immunity against HSV in the liver is not entirely surprising. Indeed, these findings are consistent with type I IFNs being essential for protective immunity against HSV-1 in the central nervous system, as demonstrated by the occurrence of herpes simplex encephalitis (HSE) due to inherited deficiencies affecting either the production or the response to type I IFNs. Testing for AAN-I-IFNs should be performed in children and adults with HSE. We recently developed a quick whole blood assay to test the presence of such auto-Abs (Gervais et al., 2024b). Moreover, the first report of AAN-I-IFNs causing a severe disease concerned a single patient with a severe disseminated infection with another herpes virus, VZV. These findings were subsequently supported by the higher risk of cutaneous diseases due to both HSV and VZV in patients hospitalized for COVID-19 pneumonia due to these AAN-I-IFNs (Busnadiego et al., 2022).

The only two known genetic etiologies of FVH are AR IL-18BP (Belkaya et al., 2019) and IL-10RB (Korol et al., 2023) deficiencies, both of which have been described in patients with HAV-triggered FVH. We recently reported another multiplex family with AR IL-18BP deficiency, in which two siblings died from HAV-triggered FVH (Abd Elaziz et al., 2025). The proposed pathophysiological mechanism involves excessive production of the macrophage-activating factor type II IFN in the liver, leading to a sustained activation of macrophages and uncontrolled inflammation (Belkaya et al., 2019; Korol et al., 2023). Indeed, IL-18BP deficiency unleashes IL-18, which is an inducer of type II IFN (Belkaya et al., 2019), whereas IL-10RB deficiency blocks the activity of IL-10, a potent inhibitor of type II IFN in macrophages (Korol et al., 2023). By contrast, in the case of HSV-triggered FVH, the presence of AAN-I-IFNs might impair the control of viral replication, leading to excessive inflammation, as observed in patients with critical COVID-19 (Casanova and Anderson, 2023). Interestingly, no patient with an IEI of the type I IFN pathway or AAN-I-IFNs has been reported to suffer from both HSV-triggered FVH and encephalitis. This is intriguing, because such deficiencies severely impair the type I IFN system in all cells of an organism. This suggests that some redundant, organ-specific anti-HSV immune mechanisms may exist in some patients but not others. The mechanism underlying the incomplete penetrance of organ-specific HSV infection warrants further investigations. Additional studies are also required to determine the genetic causes of HSV-triggered FVH, which may affect type I IFNs, and to assess the contribution of AAN-I-IFNs in patients with other manifestations of herpes infections, such as skin diseases, encephalitis, and other organ-specific infections.

## Materials and methods

### Patients

All patients were recruited retrospectively.

#### American patients

The Acute Liver Failure Study Group (ALFSG) was a National Institutes of Health (NIH)–funded network that enrolled 3,364 ALF/acute liver injury (ALI) patients in a prospective registry after they were admitted to 31 tertiary centers in North America between January 1998 and August 2019. Patients enrolled met the following criteria: for ALF, any degree of hepatic encephalopathy occurring within 26 wk of symptom onset, coagulopathy defined as an international normalized ratio (INR) ≥1.5, absent cirrhosis or any prior history of underlying liver disease; for ALI, similar illness but with an INR ≥ 2.0, and no hepatic encephalopathy. Those with hepatitis B, Wilson disease, or autoimmune hepatitis that might have some degree of fibrosis were included, if their initial disease presentation met ALF criteria. Participants (ALI) or legal next of kin (ALF) provided written informed consent; 24 patients presented with ALI but all developed ALF prior to transplantation. Data included clinical histories and up to 7 days’ detailed clinical and laboratory data. All sites complied with local institutional review board requirements and adhered to the Declarations of Helsinki and Istanbul. Patient management was based on the local standard of care, with transplant candidacy determined by each center. Sera were obtained on the first seven days following study enrollment and stored at −80°C. Patient diagnoses were confirmed by a committee of senior hepatologists, in the case of viral hepatitis, by the appropriate viral serologies.

#### French patients

Patients were included based on the following inclusion criteria: no known evidence of preexisting chronic liver disease, biochemical evidence of ALF, and hepatic coagulopathy not correctable with vitamin K, defined as: INR ≥ 1.5 with clinical hepatic encephalopathy, or INR ≥ 2.0 regardless of the presence of encephalopathy and identification of viral etiology. Participants or legal representants provided written informed consent (protocol C09-18, ID-RCB:A00039-30). Data included clinical histories and detailed clinical and laboratory data.

#### Moroccan patients

Patients were admitted to the pediatric intensive care unit for suspicion of severe or critical HAV or HBV, with or without anti-HAV or anti-HBV IgM.

#### Turkish patients

Patients were included based on the pediatric ALF (PALF) criteria established by the Pediatric Acute Liver Failure Study Group in 2006. These inclusion criteria required all the following: no known evidence of preexisting chronic liver disease, biochemical evidence of ALI, and hepatic coagulopathy not correctable with vitamin K, defined as: INR ≥ 1.5 with clinical hepatic encephalopathy, or INR ≥ 2.0 regardless of the presence of encephalopathy. Participants or legal representants provided written informed consent. Data included clinical histories and detailed clinical and laboratory data.

#### Italian patients

Patients P1 and P2 were recruited into a research protocol ongoing at Fondazione IRCCS Policlinico San Matteo (San Matteo Research Hospital), Pavia, Italy, aiming to define the prevalence of auto-Abs neutralizing type I IFNs in individuals with autoimmune conditions. Patients are recruited into the rheumatological department. Inclusion criteria include connective tissue disorders (SLE, systemic sclerosis, undifferentiated connective tissue disorder, or myositis), with or without a history of life-threatening viral infection. These two patients were the only patients admitted for HSV-FVH in this research protocol. All patients signed a written informed consent. Detailed clinical data, including demographics, information on autoimmune and infectious history and familiar recurrence are recorded for all patients. The cohort currently consists of 168 patients. P1 and P2 were recruited according to inclusion criteria for HLA-B27 spondyloenthesoarthritis (P1), and for arthritis, antinuclear antibodies and anti-smooth muscle antibodies (P2), and were the only two patients in the whole cohort with a known history of FVH.

### Luciferase reporter assay

The blocking activity of anti-IFN-α2, anti-IFN-ω, and anti-IFN-β auto-Abs was determined with a reporter luciferase assay, as previously described (Bastard et al., 2021a).

### Detection of auto-Abs by ELISA

ELISA was performed as previously described (Gervais et al., 2023; Puel et al., 2008).

### Detection of anti-HSV-1 antibodies by ELISA

Anti-HSV-1 IgG was measured in patient serum and plasma samples by ELISA (ab108738; Abcam) according to the manufacturer’s instructions. IgG positivity was calculated based on a negative, a positive and a cut-off control analyzed together with the patient samples. Samples with ELISA absorbance values within ±10% of the absorbance value for the cut-off control were considered inconclusive, samples with absorbance value > cut-off +10% were considered positive for anti-HSV-1 IgG, and samples with absorbance value < cut-off of 10% were considered negative for anti-HSV-1 IgG.

### Protein array

Detection of auto-Abs with protein arrays (HuProt from CDI Laboratories) was performed as previously described (Le Voyer et al., 2023).

### Statistical analysis

ORs and P values for the effect of auto-Abs neutralizing each type I IFN in patients with FVH were estimated relative to healthy individuals from the general population and adjusted for age in three categories (≤30, 30–50, >50 years) by Firth’s bias-corrected logistic regression, as implemented in the logistf package of R software, due to low number of auto-Ab carriers for some types of IFN. Where relevant, statistical test results are indicated in the corresponding figures. ns, not significant, *P < 0.05, ***P < 0.001, and ****P < 0.0001.

### Online supplemental material

Fig. S1 shows detection of auto-Abs by ELISA and HuProt.

## Data Availability

All data supporting the findings of this study are available within the main text and supplemental material and from the corresponding author upon request.
